# Pathophysiology and Therapeutic Potential of NADPH Oxidases in Ischemic Stroke-Induced Oxidative Stress

**DOI:** 10.1155/2021/6631805

**Published:** 2021-03-09

**Authors:** Jinnan Duan, Shiqi Gao, Sheng Tu, Cameron Lenahan, Anwen Shao, Jifang Sheng

**Affiliations:** ^1^State Key Laboratory for Diagnosis and Treatment of Infectious Diseases, National Clinical Research Center for Infectious Disease, Collaborative Innovation Center for Diagnosis and Treatment of Infectious Diseases, The First Affiliated Hospital, College of Medicine, Zhejiang University, Hangzhou, Zhejiang, China; ^2^Department of Neurosurgery, Second Affiliated Hospital, School of Medicine, Zhejiang University, Hangzhou, China; ^3^Center for Neuroscience Research, Loma Linda University School of Medicine, Loma Linda, CA, USA; ^4^Burrell College of Osteopathic Medicine, Las Cruces, NM, USA

## Abstract

Stroke is a leading cause of death and disability in humans. The excessive production of reactive oxygen species (ROS) is an important contributor to oxidative stress and secondary brain damage after stroke. Nicotinamide adenine dinucleotide phosphate (NADPH) oxidase, an enzyme complex consisting of membrane subunits and cytoplasmic subunits, regulates neuronal maturation and cerebrovascular homeostasis. However, NADPH oxidase overproduction contributes to neurotoxicity and cerebrovascular disease. NADPH oxidase has been implicated as the principal source of ROS in the brain, and numerous studies have shown that the knockout of NADPH exerts a protective effect in the model of ischemic stroke. In this review, we summarize the mechanism of activation of the NADPH oxidase family members, the pathophysiological effects of NADPH oxidase isoforms in ischemic stroke, and the studies of NADPH oxidase inhibitors to explore potential clinical applications.

## 1. Introduction

Ischemic stroke is by far the most common type of stroke [1] and poses a significant global burden on healthcare. While mortality has improved due to improvements in acute care, poststroke cognitive impairment is an increasing sequela that may eventually develop into dementia [2], and the high prevalence of depression among stroke survivors [3] remains a striking repercussion of stroke, not only with respect to the individual patient but also with respect to the caregivers and healthcare systems as well. Thrombolysis and endovascular therapy are mainstays of contemporary acute stroke treatment [4]. However, there are drawbacks, as these approaches suffer from risks, technical challenges, and contraindications [5]. As such, there is a need for additional and adjunctive tools to treat stroke and improve patient outcomes beyond the prevention of mortality.

Research has demonstrated that oxidative stress is closely related to ischemic stroke sequelae. Oxidative stress is a component of ischemia-reperfusion injury; increasing evidence has demonstrated that excessive production of ROS is closely correlated with oxidative stress. The excessive generation of ROS is also thought to arise from a series of reasons including mitochondrial electron transport chain (ETC) dysfunction, induction of cyclooxygenases (COXs), increased expression of NADPH oxidase, N-methyl-D-aspartate (NMDA) receptor stimulation, and ceramide elevation after stroke [6–8]. ROS overabundance not only leads to cellular component destruction and cell death through cellular effects, including protein destruction, lipid peroxidation, DNA damage, and intracellular calcium surge [7], but also affects vascular tone, platelet activity, and endothelial cell permeability, leading to blood-brain barrier (BBB) dysfunction [7], and the accumulation of amyloid in brain tissue due to oxidative stress injury after stroke triggers a neurodegenerative response, ultimately leading to neuronal dysfunction, even Alzheimer's disease (AD) [9–11]. Therefore, oxidative stress biomarkers may be the potential diagnostic biomarker of stroke, such as salivary glutathione (GSH), 8-hydroxydeoxyguanosine (8-OHdG), and malondialdehyde (MDA). Interestingly, a study found that salivary GSH has high sensitivity and specificity differentiates different stages of dementia severity, and additional studies demonstrated that high serum levels of MDA and 8-OHdG are associated with early cognitive impairment after stroke; these redox biomarkers may be used as noninvasive biomarkers of cognitive impairment in the future [2, 12, 13]. Recent studies found that the neuroprotective effects of Nrg1, ezetimibe, and PNGL reduce the ROS levels through specific signaling pathways [14–16]. However, the NADPH oxidase, also known as NOX, is an important component of ROS production in stroke [17, 18]. Currently, there are seven known NOX members in the NADPH family, including NOX1-5 and dioxygenase (DUOX1-2, also known as NOX6-7) [19]. Only NOX1, NOX2, NOX4, NOX5, and their cytoplasmic activator proteins are expressed in neurovascular units and produce a small amount of ROS to maintain the normal physiological activity of the central nervous system [19–26]. Researchers indicated that the ROS level was increased in the brain tissue after stroke onset, and NOX inhibitors exert a neuroprotective effect [27, 28].

## 2. NADPH Oxidases and Their Inhibitors in Ischemic Stroke

The NADPH oxidase family produces the transmembrane proteins responsible for transporting an electron from cytosolic NADPH across biological membranes, which reduces oxygen to superoxide anion [29]. NADPH oxidase is the only oxidase that produces ROS. Regarding the central nervous system, NADPH oxidase-derived ROS are necessary for normal brain function, including neuronal differentiation and neuronal signaling, but overproduction of ROS contributes to nervous system disease. The NOX paralogues have been detected in the central nervous system (CNS). mRNA for *NOX1*, *NOX2*, *NOX4*, *p67phox*, and *p47phox* have been identified in rat basilar artery endothelial cells, but NOX1 and NOX4 are also expressed at the protein level [30, 31]. Previous *in vitro* studies have reported that NOX2 and NOX4 are expressed in neurons, astrocytes, and microglia and that NOX1 is expressed in microglia [29, 32]. Activated microglia and peripheral leukocytes have been found in the setting of ischemic stroke, and they produce superoxide through NOX. Moreover, NOX2 deletion in the circulation reportedly led to better outcomes compared to NOX2 deletion in the brain [33]. Activated microglia can also promote the release of inflammatory factors, such as IL-1*β*, TNF-*α*, CCL2, and CCL3. Additionally, NOX2 inhibition reduces the activation and proliferation of microglia, the expression of CCL2 and CCL3, the formation of IL-1 receptors on the functional membrane, and the activation of IL-1*β*-dependent NF-*κ*B, thereby reducing oxidative stress and inflammation [34]. Few studies have demonstrated the protective effects of NOX4 and NOX5 deficiency in experimental stroke, but the protective mechanism remains unclear. Currently, the role of NOX1 in ischemic stroke is controversial. Therefore, more experiments are necessary to further elucidate the role of NOX in stroke. In recent years, there are numerous studies exploring the subtype selectivity, mechanism of action, and specificity of new small molecule NADPH enzyme inhibitors, which are summarized below.

## 3. Activation of NOX in Ischemic Stroke

### 3.1. Activation of NOX2 in Ischemic Stroke

**Figure 1:**
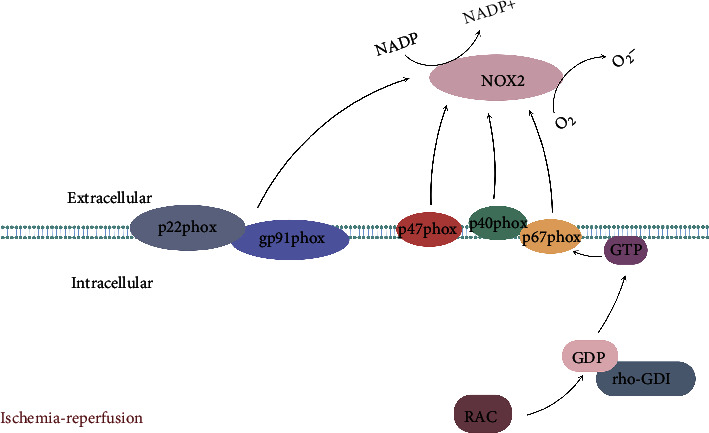
Schematic illustration of the activation mechanism of NOX2. NOX2 activation is mediated by the assembly of four cytoplasmic proteins (p67phox, p40phox, p47phox, and Rac2) with two transmembrane proteins (gp91phox and p22phox), and the activated NOX2 catalyzes oxygen to superoxide through NADPH.

NOX2 is a multicomponent enzyme system including four cytoplasmic proteins (p67phox, p40phox, p47phox, and Rac2) and two transmembrane proteins (gp91phox and p22phox). gp91phox and p22phox constitute flavocytochrome b558, which is the catalytic core of NOX2 [35]. In the hypoxic state, p47phox, p67phox, and p40phox are phosphorylated and migrated to the plasma membrane, while Rac2 converts its GDP into GTP, dissociates from rho-GDI, migrates to the membrane, and directly binds to p67phox [36]. The conformational change of p67phox is induced, and the interaction with NOX2 is promoted [37]. Then, cytochrome b558 is activated through the activation domain of p67phox and Rac2 [36]. These components combine to form enzyme complexes and become active. The activated NOX2 uses cytoplasmic NADPH to reduce oxygen to create superoxides (Figure 1).

### 3.2. Activation of NOX1 in Ischemic Stroke

**Figure 2:**
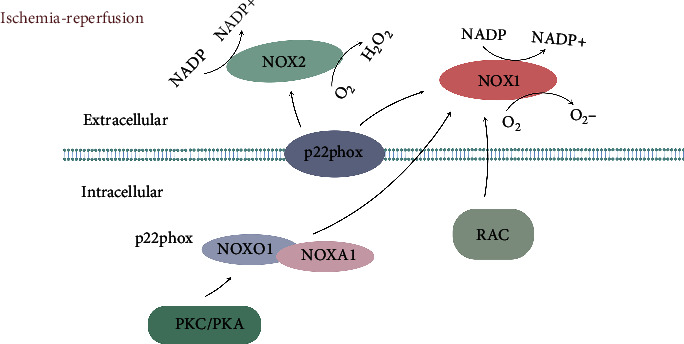
Schematic illustration of the activation mechanism of NOX1/4. NOX1 activation depends on NOXA1, NOXO1 (which is phosphorylated by PKA/PKC), Rac, and p22phox. The activated NOX1 generates superoxides at the expense of DPH; NOX4 activation requires p22phox and predominantly catalyzes the formation of hydrogen peroxide (H_2_O_2_). Abbreviations: PKC: protein kinase C; PKA: protein kinase A.

NOX1 is the first homologue of NOX2 and has two cytoplasmic subunits: NOXO1, which has the same role as p47, and NOXA1, which is similar to p67. NOX1 production requires protein kinase A (PKA) or PKC to phosphorylate NOXO1 and then interacts with NOXA1, thereby regulating NOX1 activity. In addition to these two cytoplasmic subunits, NOX1 simultaneously depends on the membrane subunit p22phox. Like NOX2, there is sufficient evidence that small GTPase Rac is involved in the regulation of NOX1 activity. Rac1 provides a primary trigger for the production of NOX1-dependent ROS, which can drastically activate NOX1-dependent ROS production [38] (Figure 2).

### 3.3. Activation of NOX4 in Ischemic Stroke

NOX4 is a transmembrane protein that requires the p22phox transmembrane protein to have biological activity. Loss of p22phox will subsequently lead to loss of activity. Compared with other NOX subtypes, NOX4 has constitutive activity without a cytoplasmic activator and can be further activated by some growth factors of TNF-*α*, angiotensin II, and PolDip2, a binding protein. NOX4 also contrasts with other NOX subtypes in that it mainly produces hydrogen peroxide (H_2_O_2_) rather than superoxide, thus mediating many downstream effects [39, 40] (Figure 2).

### 3.4. Activation of NOX5 in Ischemic Stroke

**Figure 3:**
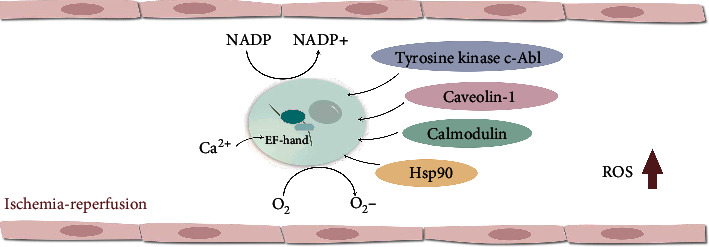
Schematic illustration of the activation mechanism of NOX5. NOX5 activation requires calcium ions by binding N-terminal EF-hand motifs to produce superoxides. The binding of Hsp90, caveolin-1, and tyrosine kinase c-Abl to NOX5 can regulate NOX5 activity and ROS production.

NOX5 is widely distributed and expressed in immune cells, as well as in the cardiovascular system, sperm, placenta, stomach, and other nonimmune systems, suggesting that the NOX5-mediated ROS process plays a vital role in the pathophysiological regulation of many systems. As such, the NOX5 activation must be strictly regulated by a variety of programs. Unique among the NADPH oxidases, NOX5 activation is concentration-dependent, as it does not require activation by NADPH subunits, and there is no glycosylation. Calcium can induce the transition from expansion to folding of the EF domain, thus binding to the dehydrogenase (DH) domain and promoting enzyme activation. Other proteins, such as calmodulin, Hsp90, caveolin-1, and tyrosine kinase c-Abl, can regulate NOX5 activity, but their functions are not strictly required [41–44] (Figure 3).

## 4. The Role of NOX in Ischemic Stroke

### 4.1. The Role of NOX2 in Ischemic Stroke

The content and activity of NOX2 are higher in cerebral arteries than systemic arteries. Therefore, the role of NOX2 in ischemic stroke has been widely discussed (Table 1). A previous study showed that 10 minutes of global brain ischemia followed by 24 hours of reperfusion has resulted in the increased expression of NOX2 and ROS [45]. Chen et al. reported that in gp91-deficient or apocynin-treated mice that had undergone 75 minutes of ischemia and 24 h of reperfusion, the absence of *NOX2* had a protective effect in ischemia-reperfusion, compared with wild-type (WT) mice. The lack of *NOX2* in this animal model reduced the ROS-mediated calcium ion-induced spectrin cleavage and the neutrophil-mediated inflammation. They also found that following reperfusion, the cerebral infarction volume of gp91 knockout mice or those treated with apocynin was 46%-50% less than that of WT mice after 72 h of reperfusion, which further indicated that the inhibition or deficiency of NOX2 induced a neuroprotective effect rather than delaying the progression of postischemic brain injury [46]. In a study by De Silva et al., NOX2-deficient mice that underwent 0.5 h of cerebral ischemia and 23.5 h of reperfusion were compared with wild-type mice, and they found that *NOX2* deficiency was neuroprotective in the early stage of cerebral ischemia [47]. Tang et al. used bone marrow chimeric animals and induced cerebral ischemia for 2 h and reperfusion for 24 h. They had similar results in which *NOX2* deficiency had a protective effect on long-term cerebral ischemia [33]. Such efforts provide evidence that NOX2 inhibitors have protective effects over various stages of cerebral ischemia-reperfusion. As such, the treatment time window is relatively wide. Furthermore, CK2 [48], proinflammatory cytokines, endothelin-1, angiotensin [49], and NMDA receptors have also been found to be elevated in the poststroke cerebral environment and may affect NOX2 expression. It is therefore important to better characterize the mechanisms that may lead to increased levels of NOX2-dependent superoxide. Further studies could also shed light on contradicting evidence, such as a study conducted by Kleinschnitz et al., wherein *NOX2* deficiency had no effect in a model of cerebral ischemia/reperfusion [50]. Generally speaking, most studies tend to corroborate the putative protective effect of *NOX2* deficiency in ischemic stroke. As such, NOX2 may be a potential new vascular and neuroprotective target in treating stroke.

### 4.2. The Role of NOX1 in Ischemic Stroke

Although the role of NOX1-produced ROS is reportedly found in an increasing number of diseases, including atherosclerosis, hypertension, neurodegenerative disease, inflammation, and cancer [51, 52], very few studies have explored NOX1 in ischemic stroke (Table 1). Kahles et al. found that the knockout of *NOX1* could reduce the volume of cerebral infarction and improve the recovery of neurological function in a rat model of ischemia-reperfusion. Moreover, in permanent cerebral ischemia (more than 2 h), wild-type mice and NOX1-deficient mice have similar cerebral infarction volumes [18], suggesting that the effect of NOX1 may be time-sensitive. Choi et al. conducted a study pertaining to the peri-infarct area and found that at 14 days of postischemia, *NOX1* knockout rats had higher neuronal survival rates, which was explained by reduced neuronal apoptosis and astrocytic activation, as well as progenitor cells that then differentiated into neurons. In addition, this is the first study to find that the levels of NOX1 and Rac1 proteins in peri-infarct neurons were significantly increased, resulting in superoxide production and DNA oxidative damage in this area [23]. NOX1-mediated oxidative damage may be the main factor influencing neurodegeneration in the peri-infarct region. In the peri-infarct area, the *NOX1* knockdown can reduce infarction size and activation of astrocytes [23]. As the *NOX1* knockdown has been shown to improve survival of peri-infarct tissue, NOX1 may become a potential therapeutic target poststroke. Conversely, Jackman et al. found that angiotensin II was required to stimulate NOX1 to increase the production of superoxide dismutase in cerebral arteries. They found no difference in total or subcortical infarct volume between *NOX1* knockout mice and wild-type mice. Moreover, cortical infarct volume in *NOX1* knockout mice was four times higher than that in wild-type mice, suggesting that NOX1 could be protective in limiting the development of cortical infarction after cerebral ischemia [53]. At present, the exact role of NOX1 in ischemia-reperfusion injury is still unclear, and more research is necessary to explore the mechanism of NOX1 in ischemic stroke.

### 4.3. The Role of NOX4 in Ischemic Stroke

NOX4 is specifically involved in cerebral ischemia. Interestingly, it is not involved in cardiovascular and peripheral vascular ischemia. After ischemic stroke, NOX4 is mainly expressed on endothelial cells, neurons, and smooth muscle cells of the central nervous system. Endothelial NOX4 can destroy the blood-brain barrier, leading to BBB leakage, whereas neuronal NOX4 can directly lead to autotoxicity and apoptosis (Table 1). A common sequela of NOX4-induced brain injury is the formation of ROS. Moreover, NOX4 is the main source of peroxidation produced by pericytes. Although pericytes have a relatively small impact on diseases and this model, they reportedly play a double-sided role in ischemic stroke. More research is needed to clarify this role and to determine the different subtypes and functions of pericytes. There is evidence to suggest that inhibiting the expression of NOX4 in pericytes may be a treatment strategy for ischemic stroke [22, 54]. In human studies of the blood-brain barrier, after 6 h of hypoxia and 24 h of reoxygenation, NOX4 was found to produce harmful effects during the subacute phase of ischemia-reperfusion (4-6 h after stroke). Pharmacological NOX4 inhibitors have been shown to be protective in the blood-brain barrier in mice [20]. In another study, which used different ages and genders of mice, as well as a different stroke model and ischemic timings (in accordance with STAIR guidelines) to continuously evaluate the functional defects seven days after stroke, it was found that NOX4 inhibition significantly reduced mortality after stroke [50]. Furthermore, no adverse complications, such as cerebral hemorrhage, occurred during NOX4 inhibition [50]. NOX4 inhibitors can better reduce oxidative stress, brain cell damage, and neuronal apoptosis in both the short- and long-term brain injury models. Additionally, NOX4 inhibitors may be safe and consistently effective [50]. More recently, Vallet et al. continuously monitored the expression of NOX4 mRNA in the cortex of mice with focal permanent cerebral ischemia, which increased in 24 h after ischemia, peaked in 7-15 days, and decreased in 30 days [55]. This study showed that NOX4 not only participated in neuronal damage in the early stage but also participated in neovascularization in the repair stage [55]. Therefore, NOX4 plays different roles in different stages of ischemia-reperfusion, and the ultimate benefits of NOX4 inhibitors on ischemic stroke warrant further research and exploration.

### 4.4. The Role of NOX5 in Ischemic Stroke

Although NOX5 is highly upregulated in human diseases, it does not exist in rodents. Therefore, the study of NOX5 in traditional mouse or rat disease models has been limited. For the first time, Casas et al. produced mice that express human NOX5 in endothelial cells and defined the previously unknown role of NOX5 in cerebral infarction [20] (Table 1). In this study, *NOX5*-KI mice were subjected to 1 hour of MCAO and 24 hours of reperfusion. Compared with wild-type mice, the production of ROS in the brain of *NOX5*-KI mice was significantly increased, leading to BBB leakage and impaired nerve function, indicating that NOX5-dependent ROS formation is related to worsening prognosis. In another study by Casas et al., they explored the role of NOX5 in the peripheral vasculature. Their findings were similar to NOX4 in that the role of NOX5 in ischemic stroke is brain-specific [20]. In an *in vitro* study, they demonstrated that after a 15-minute period of ischemia, reoxygenation or calcium overload led to increased brain ROS levels in a NOX5-dependent manner [20]. In addition, Casas et al. found that NOX5 inhibitors could prevent the leakage of human microvascular endothelial cells after acute reoxygenation only at the early stage, but not at 20 minutes after reoxygenation, which was consistent with the immediate calcium influx during reoxygenation injury [20]. However, more studies are currently needed regarding NOX5 in ischemic stroke. From the few published experimental results, NOX5 is the intermediate link between the calcium surge and the BBB destruction, and it plays a role in the acute phase of stroke. Inhibition of NOX5 may reduce ischemic injury and exert a neuroprotective effect.

## 5. New Small-Molecule NADPH Oxidase Inhibitors in the Treatment of Ischemic Stroke

Numerous studies using disease animal models have reported that NADPH oxidases are associated with oxidative tissue injury in various diseases, such as diabetic nephropathy [56], cancer [57], pulmonary fibrosis [58], liver fibrosis [59], atherosclerosis [60], and ischemic stroke [20], and NADPH inhibitor treatment can attenuate the tissue injury mentioned above. Recent research studies have revealed that new small-molecule NADPH oxidase inhibitors can reduce ROS production and inhibit brain tissue damage after ischemic stroke, of which beneficial effects are dependent upon the treatment duration and drug-specific [28]. Currently, all studied small-molecule inhibitors blocked different NOX isoforms [28]; thus, developing specifically targeted NOX inhibitors may be a potential direction for further study of ischemic stroke treatment.

### 5.1. VAS2870

VAS2870 is a pan-NOX inhibitor that plays a beneficial role in various preclinical disease models, such as stroke (Table 2), Alzheimer's disease [61], and pulmonary hypertension [62]. Kleinschnitz et al. used VAS2870 in an experimental model of stroke and found that it has a protective effect in ischemic brain injury, thereby improving neurological function [28, 50]. Casas et al. also observed the neuroprotective effect of VAS2870 in hippocampal slices *in vitro* [22]. Other recent studies report that VAS2870 can inhibit all NOX subtypes except NOX3 [28, 63]. It has been shown that VAS2870 has a strong inhibitory effect on NOX2 (IC50: 0.7 *μ*M), but NOX1 and NOX4 were suppressed in the lower micromolar range compared to the NOX5 [28, 63]. Unfortunately, *in vivo* application of VAS2870 is limited as a result of the poor solubility and strict concentration requirements of the NOX subtypes [28, 63].

### 5.2. M13

M13 is a new compound and is currently the best relative NOX4-selective inhibitor of its kind. Recent studies have shown that M13 inhibits NOX4 (IC50: 0.01 *μ*M) 200 times more than NOX1 (IC50: 0.2 *μ*M), while having almost no effect on NOX2 or NOX5. In an *in vitro* human BBB model, M13 was added 6 h after cerebral ischemia, and a neuroprotective effect was observed after 24 h of reoxygenation [28]. In another study of human brain microvascular endothelial cells treated with hypoxia and reoxygenation, M13 could protect BBB integrity when added in the early or late stage [20]. At present, these findings support the application of this new NOX4-selective inhibitor in diseases, such as stroke (Table 2).

### 5.3. GKT136901 and GKT137831

GKT136901 and GKT137831, developed by GenKyoTex (GenKyoTex SA, Plan-les-Ouates, Switzerland), are generally considered to be specific dual NOX1/NOX4 inhibitors. GKT compounds effectively inhibit NOX1, NOX4, and NOX5 in the three-digit nanomolar range and NOX1, NOX4, and NOX2 in the range of micromoles [19]. Pharmacological verification tests of GKT have shown that it interferes with a variety of determination reagents [28]. It has also been observed that GKT136901 can effectively scavenge peroxynitrite [28] and H_2_O_2_ [64]. These findings complicate the interpretation of results of GKT to date. GKT has excellent pharmacokinetic characteristics and oral bioavailability *in vivo* and has been widely used in animal models of various diseases, such as stroke [22] (Table 2), acute respiratory distress syndrome [65], liver fibrosis [66], diabetic nephropathy [67], and heart failure [68]. In *in vitro* studies exploring the effects of GKT136901 in hypoxia-reoxygenation human BBB and hippocampal slices, GKT136901 has demonstrated neuroprotective effects and improved BBB stability [22]. At present, GKT137831 is the only NOX inhibitor that has passed preclinical development to enter phase II clinical trials of diabetic nephropathy. GKT may become the most promising clinical candidate, but its other action mechanisms warrant exploration.

### 5.4. ML090

ML090 is a pan-NOX inhibitor. Its IC50 values, with respect to NOX1, NOX4, and NOX5, are similar. Some studies have confirmed that ML090 is protective in rabbit models of vascular dysfunction [69]. This therapeutic effect has been attributed to the inhibition of NOX1, but the inhibitory effects of NOX4 and NOX5 cannot be ruled out yet [69]. Moreover, ML090 has been found to prevent the leakage of human microvascular brain endothelial cells after acute reoxygenation due to the mechanism of relative inhibition of NOX5 [20] (Table 2). The efficacy of ML090 suggests that it may be a preventive drug treatment option for ischemic stroke or that it may have applications in reperfusion therapy (thrombolysis and intravascular therapy).

### 5.5. ML171

ML171 is considered an effective NOX1 inhibitor, and many preclinical studies have confirmed that it plays a beneficial role in hypertension [70], diabetes [71], and cancer [72]. However, it has recently been shown that ML171 is not absolutely NOX1-selective and inhibits NOX4 and NOX5 at slightly higher concentrations. In an *in vitro* study of the human blood-brain barrier, the neuroprotective effect of ML171 was observed after 24 h of reoxygenation in the subacute phase of cerebral ischemia [28] (Table 2). These findings suggest that ML171 could have applications in different periods of cerebral ischemia-reperfusion and may become a therapeutic drug for ischemic stroke.

## 6. Conclusion

In ischemic stroke, oxidative stress can promote disease progression even after reperfusion is achieved. As such, it is thought that reducing oxidative stress could improve patient outcomes. To date, the clinical use of nonselective ROS scavenging antioxidants has not achieved meaningful results in improving long-term disability-related outcomes of ischemic stroke. In this light, it is urgent to find effective, targeted drugs to improve the prognosis of these patients. NADPH oxidase is the main producer of ROS and is widely distributed in the central nervous system. Following ischemic stroke, the expression of NOX subtypes increases sharply in cerebral vessels and neurons. Inhibiting the activity and expression of NOX may be an important starting point for ameliorating oxidative stress in ischemic stroke, although their precise roles have yet to be fully elucidated. A variety of NOX isomer inhibitors have been shown to improve brain injury and neurological function after stroke in preclinical disease models. However, the structures of NOX subtypes are similar, and the specificities of NOX inhibitors presently under investigation are insufficient to realize homotype selection. Future studies should focus on developing inhibitors that target specific NOX homologues, which may deepen our understanding of their various roles at different stages of ischemic stroke.

## Figures and Tables

**Table 1:** The structure of NADPH oxidase and the expression and role of NOX in the brain of the cerebral ischemia-reperfusion model.

NOX subtype	Neurovascular unit	NOX subunit	Experimental model	Ischemia time	Reperfusion time	Oxidation product	Effect	References
NOX2	Astrocytes, neurons, endothelial cell, vascular smooth muscle, microglial	gp91phox p22phox p67phox p47phox Rac	NOX2 KO mice	1.25 h	24 h/72 h	O_2_^−^	+	Chen, H (2011) [34]
			NOX2 KO mice	0.5 h	23.5 h	O_2_^−^	+	De Silva, T.M (2011) [47]
			Mosaicism NOX2 KO mice	2 h	24 h	O_2_^−^	+	Tang, X.N (2011) [33]
			NOX2 KO mice	24 h	/	O_2_^−^	/	Kleinschnitz, C (2010) [50]
NOX1	Astrocytes, neurons, microglial, endothelial cell	NOXO1 NOXA1 p22phox Rac	NOX1 KO mice	1 h		O_2_^−^	+	Kahles, T (2007) [24]
			NOX1 knockdown mice	14 d/28 d	/	O_2_^−^	+	Choi, D (2015) [23]
			NOX1 KO mice	0.5 h	23.5 h	O_2_^−^	-	Jackman, K.A (2009) [53]
			NOX1 KO mice	More than 2 h	22 h	O_2_^−^	/	Kahles, T (2007) [24]
NOX4	Vascular smooth muscle, pericytes, astrocytes, neurons	p22phox	*In vitro* model of BBB	6 h	24 h	H_2_O_2_	+	Casas, A.I (2019) [20]
			NOX4 KO mice	1 h/1.5 h	24 h	H_2_O_2_	+	Kleinschnitz, C (2010) [50]
			NOX4 KO rat	7 d	/	H_2_O_2_	+	Kleinschnitz, C (2010) [50]
			Permanent brain ischemia model in mice	30 d	/	H_2_O_2_	-	Vallet, P (2005) [55]
NOX5	Endothelial cell	/	NOX5-KI mice	1 h	24 h	O_2_^−^	-	Casas, A.I (2019) [20]
			HBMECs expressing NOX5	6 h	24 h	O_2_^−^	-	Casas, A.I (2019) [20]

Abbreviations: ROS: reactive oxygen species; NOX: nicotinamide adenine dinucleotide phosphate oxidase; O_2_^−^: superoxide anion radical; H_2_O_2_: hydrogen peroxide; KO: knockout; BBB: blood-brain barrier; HBMECs: human brain microvascular endothelial cells. +, brain protection/neuroprotection; -, brain damage/neurological dysfunction.

**Table 2:** The role of NOX inhibitors in animal models of ischemic stroke.

NOX inhibitors	Pharmacological mechanism	Tissues or cells	Ischemia time	Reperfusion time	Effect	References
VAS2870	Inhibit NOX1, NOX2, NOX4, and NOX5	Brain sections of mice	12 h	/	+	Kleinschnitz, C (2010) [50]
		Rat hippocampal brain slice	0.25 h	2 h	+	Casas, A.I (2017) [22]
M13	Inhibit NOX1 and NOX4	A human *in vitro* model of BBB	6 h	24 h	+	Dao, V.T (2020) [28]
		Primary culture of HBMECs	6 h	24 h	+	Casas, A.I (2019) [20]
GKT136901	Inhibit NOX1, NOX2, NOX4, and NOX5	Primary culture of HBMECs	6 h	24 h	+	Casas, A.I (2017) [22]
		Rat hippocampal brain slice	0.25 h	2 h	+	Casas, A.I (2017) [22]
ML090	Inhibit NOX1, NOX4, and NOX5	Primary culture of HBMECs	6 h	24 h	+	Casas, A.I (2019) [20]
ML171	Inhibit NOX1, NOX4, and NOX5	A human *in vitro* model of BBB	6 h	24 h	+	Casas, A.I (2019) [20]

Abbreviations: HBMECs: human brain microvascular endothelial cells; BBB: blood-brain barrier. +, brain protection/neuroprotection; -, brain damage/neurological dysfunction.

## Data Availability

No data were used to support this review article.
